# The 24-Hour Movement Behavior Composition and the Risk of Dementia

**DOI:** 10.1177/30495334251361293

**Published:** 2025-08-04

**Authors:** Margarita Liubetskaya, Marcus Vinicius Veber Lopes, Ian Janssen

**Affiliations:** 1 Queen’s University, Kingston, ON, Canada; 2 Children’s Hospital of Eastern Ontario, Ottawa, Canada

**Keywords:** dementia, physical activity, sedentary behavior, sleep, compositional data analysis, UK Biobank

## Abstract

**Objectives:**

This study examined the relationship between the 24-hour movement behavior composition—including sleep, sedentary time, light physical activity, and moderate-to-vigorous physical activity—and dementia risk.

**Methods:**

93,781 participants (mean age: 62 years) from the UK Biobank were studied. The average daily time spent in each movement behavior was determined using accelerometers. Incident cases of dementia were identified over an average 9.6-year follow-up. Cox proportional hazards models with compositional covariates assessed the associations of interest.

**Results:**

Relative time in moderate-to-vigorous and light physical activity were negatively associated with dementia risk while relative time in sedentary behavior was positively associated with dementia risk. Each 15 min/day reallocation of time from sedentary behavior into sleep, sedentary behavior, or physical activity reduced dementia risk by 2% to 5%.

**Conclusions:**

The time-use composition of movement behaviors across the 24-hour day influences dementia risk.

## Introduction

Dementia, a syndrome associated with a decline in cognitive abilities, is a major public health problem (Gale et al., 2018). More than 55 million people worldwide are currently affected, a number expected to rise to 152 million by 2050, with annual healthcare costs exceeding $1.3 trillion USD (Ageing and Health, 2024; Nichols et al., 2022; World Health Organization, 2023). In the absence of a cure, preventative measures are needed (World Health Organization, 2023).

Sleep, sedentary behavior (SED), light physical activity (LPA), and moderate-to-vigorous physical activity (MVPA) are movement behaviors that comprise the 24-hour day (Dumuid et al., 2020; Janssen et al., 2020; Pedišić, 2014; Pedišić & Bauman, 2015). Systematic reviews have reported that insufficient (<6 hr/day) and excessive (>9 hr/day) sleep, high SED, and low MVPA are associated with an increased risk of dementia (Blondell et al., 2014; Sabia et al., 2021; Yan et al., 2020). A major limitation of the current movement behavior and dementia literature is that most studies investigated movement behaviors in isolation, and few accounted for the compositional nature of movement behavior variables (Chaput et al., 2014; Janssen et al., 2020).

Movement behaviors are compositional variables because daily time is limited to 24 hr, with sleep, SED, LPA, and MVPA always summing to this fixed total (Dumuid et al., 2020; Janssen et al., 2020). Consequently, changes in time spent in one movement behavior necessitate compensatory changes in time spent in the others. Traditional statistical methods, which assume variable independence, are unsuitable for analyzing movement behaviors due to their inherent interdependence (Dumuid et al., 2020; Janssen et al., 2020; Pawlowsky-Glahn & Egozcue, 2006).

Compositional data analysis (CoDA) is an established branch of statistics designed for compositional data (Dumuid et al., 2020; Janssen et al., 2020). In the past decade, researchers have used CoDA to improve understanding of the relationship between movement behaviors and several health indicators (Janssen et al., 2020). Although these CoDA studies have not examined dementia, analyses based on other health indicators suggest that the overall 24-hour movement behavior composition is associated with health and that the relative proportions of time spent in individual movement behaviors are associated with health (Janssen et al., 2020). Furthermore, studies have used CoDA to estimate how reallocating time from one movement behavior to another within the 24-hour day influences health (Clarke & Janssen, 2021; Janssen et al., 2020). For instance, one study reported that reallocating 15 min/day from LPA into MVPA, while maintaining sleep and SED at a constant amount, reduces all-cause mortality risk by 14% (Clarke & Janssen, 2021).

The objectives of this study were to use CoDA to: (1) examine whether the 24-hour time-use composition comprised of sleep, SED, and different intensities of physical activity predicts the risk of dementia; and (2) estimate whether reallocating time from one movement behavior into another changes the risk of dementia.

## Materials and Methods

### Data Source and Participants

This study relied on data from the UK Biobank. This ongoing, large-scale biomedical database contains in-depth health information of over 500,000 participants from the UK aged 40 to 49 years at study entry. Baseline assessments were performed between 2006 and 2010 in 22 centres across the UK and consisted of questionnaires, face-to-face interviews, physical measurements, and collection of biospecimens (About Our Data, 2023). Between February 2013 and December 2015, participants with a valid email address were invited to wear an accelerometer for seven consecutive days (Doherty et al., 2017). From June 2013 to January 2016, participants were sent accelerometer devices in order of acceptance (Doherty et al., 2017). A total of 103,627 participants returned an accelerometer with readable accelerometer data (Walmsley et al., 2022). These participants were aged 43 to 78 years (mean 62.3 years) when they wore the accelerometer. Follow-up data were collected on participants through linkages with administrative databases (About Our Data, 2023). End of follow-up for administrative linkages for the present study was July, 2024. The follow-up length for individual participants, which reflected the difference from when they wore the accelerometer to the end of the linkage period, ranged from 5 days to 11.1 years (mean 9.6 years).

**Figure 1.**
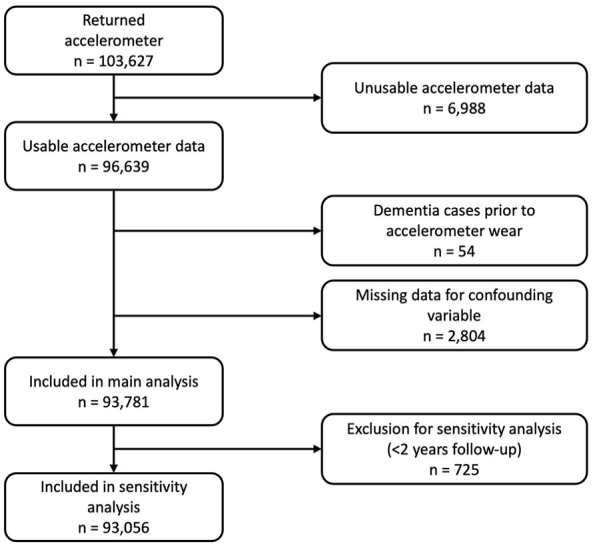
Participant flowchart.

To be included in the present study, participants were required to wear and return accelerometers with sufficient wear time (i.e., ≥3 valid days defined as not missing accelerometer data from each 1-hour period) and proper calibration (i.e., accelerometer was calibrated, <1% of the accelerometer readings were outside the dynamic range, and the average acceleration was <100 mg) (Walmsley et al., 2022). Approximately 96,639 or 93% of participants who wore accelerometers provided usable accelerometer data (Doherty et al., 2017). Participants diagnosed with dementia before the accelerometer wear period were excluded, as were participants with missing data for any confounding variable. For the sensitivity analysis, participants with dementia occurring within 2 years of the start of follow-up were excluded. This left 93,781 participants for the main analysis and 93,056 participants for the sensitivity analysis. A flowchart describing the sample and reasons for exclusion is provided in Figure 1.

### Movement Behaviors

The exposure of interest was the time-use composition of movement behaviors across the 24-hour day, which was assessed using Axivity AX3 accelerometers (Doherty et al., 2017; Walmsley et al., 2022). Participants were instructed to wear an accelerometer on the wrist of their dominant hand continuously for 7 days (Doherty et al., 2017). Devices were calibrated to local gravity to ensure similar outputs across devices under similar conditions (Doherty et al., 2017). Movement information was extracted from 100 Hz raw triaxial acceleration data using linear interpolations after calibration, removing gravity and sensor noise, and identifying wear/non-wear episodes (Doherty et al., 2017).

The summary accelerometer variables (e.g., average h/day of sleep, SED, LPA, and MVPA normalized to the 24-hour day) available in the UK Biobank database were used for our analyses (About Our Data, 2023; Walmsley et al., 2022). Raw accelerometer data processing was completed by Biobank investigators and started by algorithmically identifying missing data as unbroken episodes of at least 60 min with the SD of each axis of acceleration being <13 mg (Walmsley et al., 2022). Missing readings outside the aforementioned conditions were imputed as the average data collected in the 1 hr period on the remaining days (Walmsley et al., 2022). The next phase of accelerometer data processing consisted of identifying movement behavior intensities. These were classified using machine-learning methods described elsewhere (Doherty et al., 2017; Walmsley et al., 2022; Willetts et al., 2018).

### Dementia

All-cause dementia and specific types of dementia (Alzheimer’s disease, vascular dementia, frontotemporal dementia) were identified using an algorithmically determined outcome. Developed by the UK Biobank outcome adjudication group, this algorithm classifies disease outcomes with high positive predictive value using ICD-9/10 codes from electronic health records, including hospital admissions and records from primary and secondary healthcare providers (Wilkinson et al., 2018). Additionally, at baseline the identification of prevalent cases of dementia was supplemented with participant self-reports (Wilkinson et al., 2018). Participants with dementia at baseline or before wearing the accelerometer were removed from the analysis. For incident cases of dementia, the time to event was based on the difference between the date they wore the accelerometer and the earliest date dementia was identified in the electronic health records.

### Covariates

Potential confounding variables were chosen based on their known association with dementia and at least one movement behavior, and if they did not lie on the causal pathway. Explored confounders included age, sex, race, education, Townsend neighborhood deprivation index, disability status, smoking status, alcohol consumption, and frequency of consumption of fresh fruits, vegetables, oily fish, beef, pork, and processed meat (Walmsley et al., 2022). Confounders were identified from self-reported questionnaires and in-person interviews. The body mass index, which was obtained from measured heights and weights, was considered a mediator that lies on the causal pathway. It was not included as a confounding variable in the main analysis but was added as a confounding variable in a sensitivity analysis. The categories used for these confounding variables are presented in Supplemental Table S1.

### Statistical Analysis

Statistical analyses were performed in R version 4.4.1 (The R Project for Statistical Computing). Conventional descriptive statistics were derived. The geometric means were determined for the compositional variables as they better capture the central tendency of compositional data than arithmetic means (Pearson, 1997).

CoDA was used to assess the association between the movement behavior composition and its components with all-cause and different types of dementia. Before conducting CoDA, two zero values for LPA and 1671 zero values for MVPA were imputed using the lowest detected time in the respective behaviors in the remaining participants (0.0046 hr/day for LPA, 0.0036 hr/day for MVPA). The first step of CoDA involved transforming the movement behaviors from constrained simplex space, their natural 24-hour day form, using isometric log ratio (*ilr*) transformations. These transformations allow the movement behavior composition to be expressed as ratios of its parts, including sleep, SED, LPA, and MVPA. Performing the sing transformations generated a series of *ilr* coordinates ( $z_{i_{1}},\;z_{i_{1}},z_{i_{1}}$ ) that corresponded to the ratio of time spent in one movement behavior to the remaining behaviors. Each component of the 24-hour day corresponds to three *ilr* coordinates that capture the time distribution of the remaining components. For example, sleep was expressed as the following:

$$\begin{array}{l} z_{i_{1}}=\sqrt{\frac{3}{4}}ln\left(\frac{sleep_{i}}{\sqrt[{3}]{SED_{i}\cdot LPA_{i}\cdot MVPA_{i}}}\right),\;\\ z_{i_{2}}=\sqrt{\frac{2}{3}}ln\left(\frac{SED_{i}}{\sqrt[{2}]{LPA_{i}\cdot MVPA_{i}}}\right),and\;\\ z_{i_{1}}=\sqrt{\frac{1}{2}}\ln\left(\frac{LPA_{i}}{\sqrt{MVPA_{i}}}\right) \end{array}$$

The first coordinate, z_i1_, represents the ratio of the first listed behavior, in the above case sleep, compared to the remaining behaviors, factoring for the behavior’s interrelationship. The remaining two coordinates, z_i2_ and z_i3_, were only included to fit the regression model. Only the beta coefficient for z_i1_ is meaningful and presented in the results.

Once transformed, the movement behavior *ilr* coordinate system can be included in a traditional regression model. Cox proportional hazard regression models with incident dementia as the outcome were used. Separate models were run for each movement behavior (because they each had a different *ilr* coordinate system) for all-cause dementia and each type of dementia. The covariates in the models included the corresponding *ilr* coordinates and the confounding variables. To yield meaningful interpretations, back transformations were performed that estimated how much reallocating time from one movement behavior into another changed the risk of dementia. For example, we estimated the hazard ratio (HR) associated with removing 15 min/day from the average time spent in MVPA and adding 15 min/day to the average time spent in sleep. Because compositional data are relative, the time displacement predictions must be made in relation to a reference point (Chastin et al., 2015; Dumuid et al., 2018). The study sample geometric mean movement behavior composition was used as the reference point.

## Results

### Descriptive Characteristics at Baseline

Key descriptive characteristics of the sample are in Table 1. An extended version of the table that includes all confounding variables is in Supplemental Table S1. The average age of participants was 62.3 years. There were more females than males (56% vs 44%). The majority were white (97%), pursued a higher education (59%), and never smoked (57%). The geometric means were 9.07 hr/day (37.8% of 24 hr) for sleep, 9.54 hr/day (39.8% of 24 hr) for SED, 4.94 hr/day (20.6% of 24 hr) for LPA, and 0.45 hr/day (1.9% of 24 hr) for MVPA.

**Table 1.** Movement Behavior of 93,781 UK Biobank Participants by Key Sociodemographic and Behavioral Characteristics.

Sample or subsample	*N* (%)	Sleep (hr/day)	SED (hr/day)	LPA (hr/day)	MVPA (hr/day)
*Overall*	93,781 (100%)	8.7 (8.0, 9.5)	9.4 (8.2, 10.6)	4.9 (3.9, 6.1)	0.6 (0.3, 1.0)
*Age, years*					
<45	6,815 (7.3%)	8.61 (7.97, 9.32)	9.51 (8.19, 10.74)	4.95 (3.86, 6.19)	0.63 (0.33, 1.03)
45–49	12,193 (13%)	8.60 (7.93, 9.35)	9.56 (8.23, 10.78)	4.88 (3.83, 6.13)	0.61 (0.31, 1.02)
50–54	14,304 (15%)	8.65 (7.97, 9.42)	9.48 (8.21, 10.71)	4.90 (3.88, 6.10)	0.59 (0.29, 0.99)
55–59	18,035 (19%)	8.74 (8.06, 9.51)	9.30 (8.08, 10.49)	5.00 (3.97, 6.15)	0.58 (0.29, 1.00)
60–64	23,366 (25%)	8.82 (8.14, 9.61)	9.29 (8.12, 10.45)	4.97 (3.96, 6.07)	0.54 (0.26, 0.94)
≥65	19,068 (20%)	8.82 (8.11, 9.64)	9.48 (8.34, 10.67)	4.83 (3.81, 5.94)	0.46 (0.21, 0.83)
*Sex*					
Female	52,928 (56%)	8.77 (8.12, 9.53)	9.12 (7.96, 10.26)	5.30 (4.30, 6.41)	0.47 (0.22, 0.82)
Male	40,853 (44%)	8.67 (7.96, 9.50)	9.81 (8.57, 11.00)	4.43 (3.48, 5.53)	0.68 (0.35, 1.14)
*Race*					
White	90,774 (97%)	8.74 (8.06, 9.52)	9.40 (8.19, 10.61)	4.92 (3.89, 6.07)	0.55 (0.27, 0.96)
Other	3,007 (3.2%)	8.51 (7.72, 9.35)	9.53 (8.18, 10.73)	5.13 (4.00, 6.40)	0.49 (0.25, 0.86)
*Education*					
School leaver	7,567 (8.1%)	9.00 (8.26, 9.90)	9.12 (7.85, 10.34)	5.02 (3.97, 6.20)	0.41 (0.18, 0.78)
Further education	30,474 (32%)	8.80 (8.10, 9.60)	9.26 (8.03, 10.49)	5.07 (3.99, 6.22)	0.49 (0.23, 0.88)
Higher education	55,740 (59%)	8.67 (8.00, 9.42)	9.52 (8.32, 10.70)	4.84 (3.84, 5.97)	0.61 (0.31, 1.02)
*Smoking status*					
Never	53,664 (57%)	8.73 (8.06, 9.50)	9.37 (8.15, 10.57)	4.96 (3.92, 6.12)	0.57 (0.28, 0.97)
Past	33,688 (36%)	8.73 (8.04, 9.51)	9.43 (8.23, 10.61)	4.91 (3.90, 6.03)	0.55 (0.26, 0.97)
Current	6,429 (6.9%)	8.76 (8.04, 9.62)	9.62 (8.35, 10.92)	4.71 (3.65, 5.96)	0.45 (0.18, 0.84)
*Alcohol consumption*					
Never	5,227 (5.6%)	8.75 (8.02, 9.64)	9.32 (8.09, 10.61)	5.04 (3.89, 6.24)	0.43 (0.18, 0.82)
1–3 times a month	18,983 (20%)	8.78 (8.06, 9.60)	9.38 (8.14, 10.66)	4.98 (3.90, 6.18)	0.45 (0.20, 0.80)
1–2 days/week	23,498 (25%)	8.76 (8.07, 9.55)	9.40 (8.17, 10.58)	4.93 (3.91, 6.06)	0.55 (0.27, 0.94)
3–4 day/week	24,510 (26%)	8.71 (8.06, 9.46)	9.42 (8.23, 10.57)	4.90 (3.89, 6.02)	0.62 (0.32, 1.04)
*Vegetable intake*					
<3 servings/day	14,769 (16%)	8.74 (8.03, 9.57)	9.70 (8.44, 10.95)	4.61 (3.60, 5.81)	0.53 (0.25, 0.93)
3–4 servings/day	35,977 (38%)	8.73 (8.05, 9.51)	9.46 (8.25, 10.64)	4.86 (3.86, 6.01)	0.56 (0.28, 0.97)
5+ servings/day	43,035 (46%)	8.73 (8.05, 9.50)	9.27 (8.06, 10.46)	5.08 (4.04, 6.21)	0.55 (0.27, 0.96)
*Fruit intake*					
<3 servings/day	58,376 (62%)	8.75 (8.06, 9.54)	9.49 (8.26, 10.70)	4.83 (3.81, 5.98)	0.54 (0.26, 0.93)
3–4 servings/day	28,389 (30%)	8.71 (8.04, 9.48)	9.27 (8.08, 10.45)	5.08 (4.06, 6.22)	0.57 (0.29, 0.98)
5+ servings/day	7,016 (7.5%)	8.65 (7.97, 9.44)	9.21 (7.99, 10.45)	5.11 (4.06, 6.30)	0.61 (0.30, 1.06)

*Note*. SED = sedentary behavior; LPA = light physical activity, MVPA = moderate-to-vigorous physical activity.

### Follow-Up Information

The follow-up length for dementia ranged from 5 days to 4,051 days (11.1 years) and was 3,513 days (9.6 years) on average. During the 885,757 person-years of follow-up, 773 cases of all-cause dementia occurred with an incidence rate of 87 per 100,000 person-years. The corresponding incidence rates for Alzheimer’s disease and vascular dementia were 36 per 100,000 person-years (319 cases) and 17 per 100,000 person-years (148 cases), respectively. There were no reported cases of frontotemporal dementia.

### Associations Between 24-Hour Movement Behaviors and Dementia

Associations between the 24-hour movement behavior composition and its components with all-cause dementia, Alzheimer’s disease, and vascular dementia are presented in Table 2. The 24-hour movement behavior composition was significantly associated with all-cause dementia (*p* < .001), Alzheimer’s disease (*p* = .01), and vascular dementia (*p* = .023). The HRs for the movement behaviors determined by these regression models represent a proportional change in dementia risk associated with an increase in time spent in that behavior relative to time spent in the remaining movement behaviors. Relative time spent in SED was positively associated with all-cause dementia (HR = 1.89, 95% CI: [1.42, 2.52]), while relative time spent in LPA (HR = 0.80, 95% CI: [0.66, 0.97]) and MVPA (HR = 0.91, 95% CI: [0.86, 0.97]) were negatively associated with all-cause dementia. Relative time spent in sleep was not significantly associated with all-cause dementia (HR = 0.73, 95% CI: [0.52, 1.02]).

**Table 2.** Compositional Cox Regression Model Estimates of the Association Between Movement Behaviors and Dementia.

Dementia outcome	Model *p*-value	Sleep		SED		LPA		MVPA	
		HR [95% CI]	*p*-Value	HR [95% CI]	*p*-Value	HR [95% CI]	*p*-Value	HR [95% CI]	*p*-Value
All-cause dementia	<.001	0.73 [0.52, 1.02]	.066	1.89 [1.42, 2.52]	<.001	0.80 [0.66, 0.97]	.020	0.91 [0.86, 0.97]	.002
Alzheimer’s disease	.010	0.88 [0.52, 1.50]	.635	1.40 [0.89, 2.21]	.142	0.92 [0.67, 1.26]	.595	0.88 [0.80, 0.97]	.009
Vascular dementia	.023	0.68 [0.32, 1.44]	.314	1.90 [1.00, 3.63]	.051	0.88 [0.57, 1.36]	.566	0.88 [0.76, 1.00]	.055

*Note*. SED = sedentary behavior; LPA = light physical activity, MVPA = moderate-to-vigorous physical activity.

Although the HRs for Alzheimer’s disease and vascular dementia were similar to those observed for all-cause dementia (Table 2), the 95% CI were less precise, and none of the HRs were statistically significant. The exception was the HR for the relative time spent in MVPA for Alzheimer’s disease (HR = 0.88, 95% CI: [0.80, 0.97], *p* = .009).

### Compositional Isometric Substitution Modelling

The baseline movement behavior composition for the substitution modeling was the geometric mean of each movement behavior (9.07 hr/day of sleep, 9.54 hr/day of SED, 4.94 hr/day of LPA, and 0.45 hr/day of MVPA). Table 3 shows the HRs associated with reallocating 15 min/day from one movement behavior to another from the geometric mean. Estimates revealed an increased risk of all-cause dementia associated with reallocating 15 min/day of MVPA into sleep, SED, or LPA (HRs for 15 min/day reallocations ranged from 1.06 to 1.08). Conversely, reallocating 15 min/day from sleep, SED, or LPA into MVPA was associated with a reduced risk of all-cause dementia (HRs for 15 min/day reallocations ranged from 0.95 to 0.97). Decreasing SED by 15 min/day by reallocating that time into any of the other movement behaviors was associated with a reduced risk of all-cause dementia (HRs = 0.95–0.98), while increasing SED by 15 min/day was associated with an increased risk (HRs = 1.02–1.08). Time reallocations between sleep and LPA did not change dementia risk (HR = 1.00 for 15 min/day displacements).

**Table 3.** Estimated Change in the Hazard Ratio for Dementia Attributable to Movement Behavior Time Reallocations.

Remove 15 min/day from	Add 15 min/day to			
	Sleep	SED	LPA	MVPA
*All-cause dementia*				
Sleep	—	1.02 (1.01, 1.04)	1.00 (0.98, 1.01)	0.97 (0.95, 1.00)
SED	0.98 (0.96, 0.99)	—	0.98 (0.97, 0.99)	0.95 (0.93, 0.97)
LPA	1.00 (0.99, 1.02)	1.02 (1.01, 1.04)	—	0.97 (0.95, 1.00)
MVPA	1.06 (1.02, 1.11)	1.08 (1.04, 1.13)	1.06 (1.01, 1.11)	—
*Alzheimer’s disease*				
Sleep	—	1.01 (0.99, 1.03)	1.00 (0.98, 1.02)	0.96 (0.92, 0.99)
SED	0.99 (0.97, 1.01)	—	0.99 (0.97, 1.01)	0.95 (0.91, 0.98)
LPA	1.00 (0.98, 1.02)	1.01 (0.99, 1.03)	—	0.96 (0.92, 1.00)
MVPA	1.09 (1.02, 1.16)	1.10 (1.03, 1.17)	1.09 (1.01, 1.17)	—
*Vascular dementia*				
Sleep	—	1.02 (0.99, 1.06)	1.00 (0.97, 1.04)	0.96 (0.91, 1.01)
SED	0.98 (0.95, 1.01)	—	0.98 (0.96, 1.00)	0.94 (0.89, 0.99)
LPA	1.00 (0.97, 1.03)	1.02 (0.99, 1.05)	—	0.96 (0.90, 1.02)
MVPA	1.09 (0.99, 1.20)	1.11 (1.01, 1.22)	1.09 (0.98, 1.21)	—

*Note*. SED = sedentary behavior; LPA = light physical activity, MVPA = moderate-to-vigorous physical activity.

**Figure 2.**
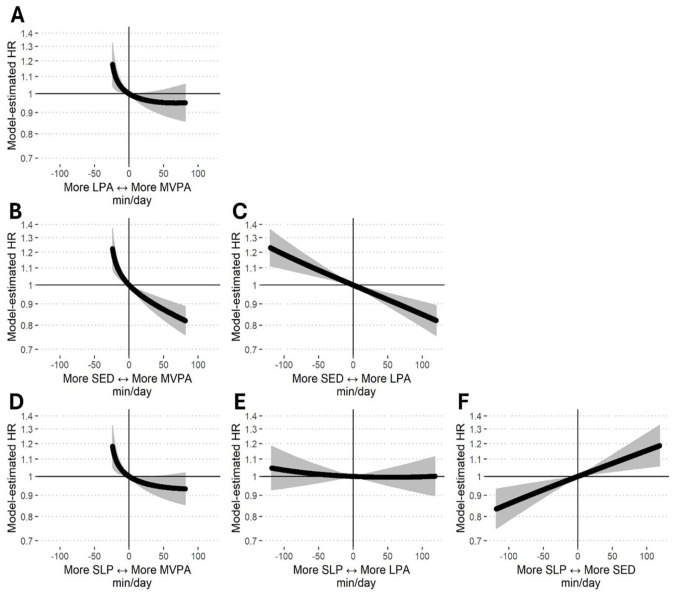
Estimated hazard ratios associated with hypothetical time reallocations from one movement behavior to another. Panel A: reallocations between LPA and MVPA, Panel B: reallocations between SED and MVPA, Panel C: reallocations between SED and LPA, Panel D: reallocations between SLP and MVPA. Panel E: reallocations between SLP and LPA, Panel F: reallocations between SLP and SED. *Note*. LPA = light physical activity; MVPA = moderate-to-vigorous physical activity; SED = sedentary behavior; SLP = sleep.

Figure 2 illustrates estimated changes in all-cause dementia risk associated with movement behavior time reallocations on a continuum. The 0 values on the *x*-axis in each panel of the figure represent the baseline for the substitution modeling, which was the geometric mean of each movement behavior. The movement behaviors on the *x*-axis represent the two behaviors involved in the time reallocation. For example, in Panel A, moving to the left of 0 represents reallocating time from MVPA into LPA while moving to the right of 0 represents relocating time from LPA into MVPA. Two key patterns are observed in Figure 2. First, time reallocations involving MVPA were curvilinear and indicated that the increased risk associated with reallocating time out of MVPA was greater than the decreased risk associated with reallocating time into MVPA (Panels A, B, and D). Second, the estimated changes in dementia risk for time reallocations between SED and LPA (Panel C) and between SED and sleep (Panel F) were approximately linear.

### Sensitivity Analysis

We re-examined the associations after including body mass index categories as an additional confounder. These results closely mirrored those of the original analysis with HRs for the individual movement behaviors differing by <0.1. The HRs for all-cause dementia were 0.72 (95% CI: [0.52, 1.01]) for sleep, 1.95 (95% CI: [1.46, 2.60]) for SED, 0.79 (95% CI: [0.65, 0.95]) for LPA, and 0.90 (95% CI: [0.85, 0.96]) for MVPA.

We also re-examined the associations after removing incident cases of dementia occurring during the initial 2 years of follow-up. For this analysis, there were 725 cases of dementia occurring over 885,112 person-years of follow-up. The HRs for sleep and SED were somewhat stronger than in the original analysis while the HRs for LPA and MVPA were largely the same as in the original analysis. The HRs for all-cause dementia were 0.63 (95% CI: [0.44, 0.89]) for sleep, 2.16 (95% CI: [1.60, 2.90]) for SED, 0.81 (95% CI: [0.66, 0.99]) for LPA, and 0.91 (95% CI: [0.85, 0.97]) for MVPA.

## Discussion

This study used CoDA to investigate the temporal relationship between the 24-hour movement behavior composition and dementia in a sample of 93,781 adults. The overall composition was associated with the risk of dementia, with positive associations observed for the relative time spent in SED and negative associations observed for the relative time spent in LPA and MVPA. Estimates suggest that dementia risk would be reduced by reallocating more time into MVPA and reallocating more time out of SED, regardless of which of the remaining movement behaviors were involved in the time reallocation.

The key findings of our study are consistent with those of a recent systematic review of studies that used CoDA to explore the association between the 24-hour movement behavior composition and health (Janssen et al., 2020). That review concluded that the 24-hour movement behavior composition was associated with a variety of health indicators and that reallocating time into MVPA and out of SED benefited health. We are unaware of other studies that used CoDA to investigate the 24-hour movement behavior composition and dementia. However, Mitchell et al. (2023) investigated the cross-sectional relationship between 24-hour movement behaviors and cognition in 4,481 adults aged 46. They found that reallocating time into MVPA from sleep (OR per 7 min/day = 1.20, 95% CI: [0.01, 2.39]), sedentary behavior (OR per 9 min/day = 1.31, 95% CI: [0.09, 2.50]), or LPA (OR per 7 min/day = 1.27, 95% CI: [0.07, 2.46]) was associated with an increased odds of high cognitive scores (Mitchell et al., 2023). Feter et al. (2023) also examined cross-sectional associations between the 24-hour movement behavior composition and cognition in adults aged 41 to 84. They observed that reallocating 5 min/day from a combination of sleep, SED, and LPA into MVPA was associated with a lower odds of poor cognitive function in sufficient sleepers (OR = 0.62, 95% CI: [0.58, 0.67]) and insufficient sleepers (OR = 0.64, 95% CI: [0.54, 0.77]) (Feter et al., 2023).

The associations between movement behaviors and dementia risk observed in epidemiological studies may be explained by several physiological effects of physical activity on the brain. MVPA enhances neurogenesis, synaptic plasticity, and cerebral perfusion in regions critical for memory and executive function (Erickson et al., 2011; Vaynman et al., 2004). These benefits are mediated by several proposed factors, including increased levels of brain-derived neurotrophic factor, which regulates synaptic strength, promotes structural changes in dendritic spines, and stimulates neurogenesis (Miranda et al., 2019). In contrast, prolonged SED is linked to impaired vascular health, increased oxidative stress, and reduced brain glucose metabolism, all of which are associated with cognitive decline and dementia risk (Franzoni et al., 2021; Gorelick et al., 2016; Iadecola et al., 2019; Wheeler et al., 2017). Evidence also suggests that reallocating time from SED or other low-intensity behaviors into MVPA amplifies neuroprotective effects by improving cardiovascular fitness, increasing cerebral perfusion, and reducing systemic inflammation (Kirk-Sanchez & McGough, 2014). These findings highlight that the epidemiological evidence of an association between movement behaviors and dementia are biologically plausible, and thereby increases confidence that these associations observed in our study are causal.

The methods and results of our study align with the principles reflected in the Canadian 24-Hour Movement Guidelines, particularly regarding time reallocation recommendations. The guidelines recommend that “Replacing sedentary behavior with additional physical activity and trading light physical activity for more moderate to vigorous physical activity, while preserving sufficient sleep, can provide greater health benefits” (Canadian 24-Hour Movement Guidelines, 2024a, 2024b). By addressing dementia, this study adds to the evidence used to inform the development of the 24-Hour Movement Guidelines. Moreover, the findings provide valuable evidence to inform dementia prevention strategies and dementia-specific public health guidelines, which are vital given that dementia has no known cure.

Our study has several limitations. For starters, the study may have been underpowered to detect small differences in risk for dementia subtypes. Accelerometers, albeit an improvement over self-reported data, are imperfect measurement tools. The wrist-worn accelerometers used in UK Biobank are susceptible to misclassifying some arm movements into higher intensities of activity. Furthermore, accelerometer data collection was limited to 3 to 7 days. This limited period may not have reflected participants’ habitual movement behavior patterns, and would have also contributed to misclassification of the movement behaviors. The two sources of misclassification likely led to attenuated HR estimates in our study.

## Conclusions

CoDA was used to provide insight into the co-dependent relationships between movement behaviors and dementia. The 24-hour movement behavior composition was associated with all-cause dementia, Alzheimer’s disease, and vascular dementia. Reallocating time from SED into any of the movement behaviors yielded a lower associated risk of dementia. Conversely, reallocating time spent in any of the movement behaviors into MVPA decreased the associated risk of dementia. Future studies should consider using CoDA to identify the optimal mixture of movement behaviors across the 24-hour day to reduce dementia risk.

## Supplemental Material

sj-docx-1-ggm-10.1177_30495334251361293 – Supplemental material for The 24-Hour Movement Behavior Composition and the Risk of Dementia Supplemental material, sj-docx-1-ggm-10.1177_30495334251361293 for The 24-Hour Movement Behavior Composition and the Risk of Dementia by Margarita Liubetskaya, Marcus Vinicius Veber Lopes and Ian Janssen in Sage Open Aging
