# Supplementary material for: The 24-Hour Movement Behavior Composition and the Risk of Dementia
Source: Sage Open Aging. 2025 Aug 4;11:30495334251361293. doi: 10.1177/30495334251361293 (PMC12322354; doi:10.1177/30495334251361293)
Supplement: sj-docx-1-ggm-10.1177_30495334251361293 – Supplemental material for The 24-Hour Movement Behavior Composition and the Risk of Dementia [file sj-docx-1-ggm-10.1177_30495334251361293.docx]

**Table S1:** Sociodemographic and movement behaviour characteristics of 93,781 UK Biobank participants

|  | **N (%)** | **Sleep (hr/day)** | **SED (hr/day)** | **LPA (hr/day** | **MVPA (min/day** |
| --- | --- | --- | --- | --- | --- |
| Overall | 93,781(100%) | 8.7 (8.0, 9.5) | 9.4 (8.2, 10.6) | 4.9 (3.9, 6.1) | 0.6 (0.3, 1.0) |
| Age, years |  |  |  |  |  |
| <50 | 6,815 (7.3%) | 8.61 (7.97, 9.32) | 9.51 (8.19, 10.74) | 4.95 (3.86, 6.19) | 0.63 (0.33, 1.03) |
| 50-54 | 12,193 (13%) | 8.60 (7.93, 9.35) | 9.56 (8.23, 10.78) | 4.88 (3.83, 6.13) | 0.61 (0.31, 1.02) |
| 55-59 | 14,304 (15%) | 8.65 (7.97, 9.42) | 9.48 (8.21, 10.71) | 4.90 (3.88, 6.10) | 0.59 (0.29, 0.99) |
| 60-64 | 18,035 (19%) | 8.74 (8.06, 9.51) | 9.30 (8.08, 10.49) | 5.00 (3.97, 6.15) | 0.58 (0.29, 1.00) |
| 65-70 | 23,366 (25%) | 8.82 (8.14, 9.61) | 9.29 (8.12, 10.45) | 4.97 (3.96, 6.07) | 0.54 (0.26, 0.94) |
| ≥70 | 19,068 (20%) | 8.82 (8.11, 9.64) | 9.48 (8.34, 10.67) | 4.83 (3.81, 5.94) | 0.46 (0.21, 0.83) |
| Sex |  |  |  |  |  |
| Female | 52,928 (56%) | 8.77 (8.12, 9.53) | 9.12 (7.96, 10.26) | 5.30 (4.30, 6.41) | 0.47 (0.22, 0.82) |
| Male | 40,853 (44%) | 8.67 (7.96, 9.50) | 9.81 (8.57, 11.00) | 4.43 (3.48, 5.53) | 0.68 (0.35, 1.14) |
| Race |  |  |  |  |  |
| White | 90,774 (97%) | 8.74 (8.06, 9.52) | 9.40 (8.19, 10.61) | 4.92 (3.89, 6.07) | 0.55 (0.27, 0.96) |
| Other | 3,007 (3.2%) | 8.51 (7.72, 9.35) | 9.53 (8.18, 10.73) | 5.13 (4.00, 6.40) | 0.49 (0.25, 0.86) |
| Body Mass Index |  |  |  |  |  |
| Normal weight  (18.5-24.9 kg/m^2^) | 37,068 (40%) | 8.74 (8.09, 9.47) | 9.05 (7.88, 10.20) | 5.21 (4.18, 6.36) | 0.65 (0.35, 1.07) |
| Overweight  (25-29.9 kg/m^2^) | 38,597 (41%) | 8.74 (8.05, 9.53) | 9.49 (8.29, 10.67) | 4.84 (3.84, 5.95) | 0.55 (0.28, 0.96) |
| Obesity class I  (30-34.9 kg/m^2^) | 13,413 (14%) | 8.72 (7.99, 9.57) | 9.90 (8.67, 11.09) | 0.55 (0.28, 0.96) | 0.40 (0.18, 0.75) |
| Obesity class II+  (≥35 kg/m^2^) | 4,703 (5.0%) | 8.68 (7.88, 9.62) | 10.39 (9.08, 11.61) | 4.29 (3.29, 5.43) | 0.23 (0.08, 0.50) |
| Physical Disability |  |  |  |  |  |
| No disability allowance | 91,075 (97.1%) | 8.73 (8.05, 9.50) | 9.39 (8.18, 10.59) | 4.94 (3.92, 6.09) | 0.56 (0.28, 0.97) |
| Disability allowance | 2,706 (2.9%) | 8.96 (8.10, 9.98) | 9.99 (8.69, 11.38) | 4.38 (3.18, 5.63) | 0.20 (0.05, 0.48) |
| Education |  |  |  |  |  |
| School Leaver | 7,567 (8.1%) | 9.00 (8.26, 9.90) | 9.12 (7.85, 10.34) | 5.02 (3.97, 6.20) | 0.41 (0.18, 0.78) |
| Further Education | 30,474 (32%) | 8.80 (8.10, 9.60) | 9.26 (8.03, 10.49) | 5.07 (3.99, 6.22) | 0.49 (0.23, 0.88) |
| Higher Education | 55,740 (59%) | 8.67 (8.00, 9.42) | 9.52 (8.32, 10.70) | 4.84 (3.84, 5.97) | 0.61 (0.31, 1.02) |
| Townsend Deprivation Index |  |  |  |  |  |
| 1^st^ quintile (-6.26 to -4.07) | 18,953 (20%) | 8.77 (8.09, 9.53) | 9.34 (8.15, 10.54) | 4.98 (3.96, 6.09) | 0.55 (0.27, 0.95) |
| 2^nd^ quintile (-4.07 to -3) | 18,912 (20%) | 8.77 (8.10, 9.52) | 9.33 (8.16, 10.52) | 4.97 (3.95, 6.11) | 0.55 (0.27, 0.96) |
| 3^rd^ quintile (-3 to -1.74) | 18,742 (20%) | 8.76 (8.09, 9.54) | 9.35 (8.14, 10.53) | 4.98 (3.95, 6.10) | 0.54 (0.26, 0.95) |
| 4^th^ quintile (-1.74 to 0.57) | 18,760 (20%) | 8.71 (8.03, 9.50) | 9.45 (8.20, 10.65) | 4.91 (3.87, 6.09) | 0.55 (0.27, 0.95) |
| 5^th^ quintile (0.57 to 10.6) | 18,414 (20%) | 8.66 (7.95, 9.48) | 9.57 (8.32, 10.81) | 4.79 (3.73, 5.99) | 0.58 (0.28, 0.98) |
| Smoking Status |  |  |  |  |  |
| Never | 53,664 (57%) | 8.73 (8.06, 9.50) | 9.37 (8.15, 10.57) | 4.96 (3.92, 6.12) | 0.57 (0.28, 0.97) |
| Past | 33,688 (36%) | 8.73 (8.04, 9.51) | 9.43 (8.23, 10.61) | 4.91 (3.90, 6.03) | 0.55 (0.26, 0.97) |
| Current | 6,429 (6.9%) | 8.76 (8.04, 9.62) | 9.62 (8.35, 10.92) | 4.71 (3.65, 5.96) | 0.45 (0.18, 0.84) |
| Alcohol Consumption |  |  |  |  |  |
| Never | 5,227 (5.6%) | 8.75 (8.02, 9.64) | 9.32 (8.09, 10.61) | 5.04 (3.89, 6.24) | 0.43 (0.18, 0.82) |
| 1-3 times a month | 18,983 (20%) | 8.78 (8.06, 9.60) | 9.38 (8.14, 10.66) | 4.98 (3.90, 6.18) | 0.45 (0.20, 0.80) |
| 1-2 days/week | 23,498 (25%) | 8.76 (8.07, 9.55) | 9.40 (8.17, 10.58) | 4.93 (3.91, 6.06) | 0.55 (0.27, 0.94) |
| 3-4 day/week | 24,510 (26%) | 8.71 (8.06, 9.46) | 9.42 (8.23, 10.57) | 4.90 (3.89, 6.02) | 0.62 (0.32, 1.04) |
| Almost daily or daily | 21,563 (23%) | 8.70 (8.02, 9.44) | 9.44 (8.23, 10.65) | 4.88 (3.88, 6.02) | 0.61 (0.31, 1.04) |
| Vegetable Intake |  |  |  |  |  |
| <3 servings/day | 14,769 (16%) | 8.74 (8.03, 9.57) | 9.70 (8.44, 10.95) | 4.61 (3.60, 5.81) | 0.53 (0.25, 0.93) |
| 3-4.9 servings/day | 35,977 (38%) | 8.73 (8.05, 9.51) | 9.46 (8.25, 10.64) | 4.86 (3.86, 6.01) | 0.56 (0.28, 0.97) |
| 5+ servings/day | 43,035 (46%) | 8.73 (8.05, 9.50) | 9.27 (8.06, 10.46) | 5.08 (4.04, 6.21) | 0.55 (0.27, 0.96) |
| Fruit Intake |  |  |  |  |  |
| <3 servings/day | 58,376 (62%) | 8.75 (8.06, 9.54) | 9.49 (8.26, 10.70) | 4.83 (3.81, 5.98) | 0.54 (0.26, 0.93) |
| 3-4.9 servings/day | 28,389 (30%) | 8.71 (8.04, 9.48) | 9.27 (8.08, 10.45) | 5.08 (4.06, 6.22) | 0.57 (0.29, 0.98) |
| 5+ servings/day | 7,016 (7.5%) | 8.65 (7.97, 9.44) | 9.21 (7.99, 10.45) | 5.11 (4.06, 6.30) | 0.61 (0.30, 1.06) |
| Oily Fish Intake |  |  |  |  |  |
| Never | 8,674 (9.2%) | 8.73 (8.02, 9.55) | 9.46 (8.17, 10.71) | 4.89 (3.80, 6.12) | 0.53 (0.24, 0.94) |
| <1 time/week | 31,949 (34%) | 8.72 (8.03, 9.51) | 9.48 (8.24, 10.67) | 4.87 (3.85, 6.03) | 0.56 (0.27, 0.96) |
| 1 time/week | 36,652 (39%) | 8.74 (8.07, 9.50) | 9.36 (8.17, 10.56) | 4.96 (3.94, 6.10) | 0.55 (0.27, 0.96) |
| 2+ times/week | 16,506 (18%) | 8.75 (8.06, 9.53) | 9.34 (8.15, 10.53) | 4.97 (3.94, 6.11) | 0.56 (0.28, 0.97) |
| Beef Intake |  |  |  |  |  |
| Never | 10,578 (11%) | 8.70 (8.01, 9.47) | 9.22 (7.97, 10.40) | 5.12 (4.06, 6.27) | 0.62 (0.32, 1.04) |
| <1 time/week | 44,764 (48%) | 8.73 (8.05, 9.51) | 9.42 (8.22, 10.62) | 4.91 (3.90, 6.06) | 0.55 (0.27, 0.96) |
| 1 time/week | 28,571 (30%) | 8.74 (8.06, 9.52) | 9.43 (8.21, 10.64 | 4.90 (3.87, 6.04) | 0.54 (0.26, 0.95) |
| 2+ times/week | 9,868 (11%) | 8.78 (8.05, 9.58) | 9.47 (8.22, 10.73) | 4.87 (3.80, 6.04) | 0.50 (0.23, 0.90) |
| Pork Intake |  |  |  |  |  |
| Never | 15,060 (16%) | 8.71 (8.03, 9.50) | 9.25 (8.03, 10.45) | 5.07 (4.03, 6.23) | 0.58 (0.28, 1.00) |
| <1 time/week | 55,913 (60%) | 8.72 (8.05, 9.50) | 9.42 (8.22, 10.63) | 4.91 (3.89, 6.05) | 0.56 (0.27, 0.96) |
| 1 time/ week | 20,035 (21%) | 8.77 (8.08, 9.55) | 9.45 (8.20, 10.65) | 4.88 (3.84, 6.03) | 0.53 (0.25, 0.93) |
| 2+ times/week | 2,773 (3.0%) | 8.68 (7.94, 9.53) | 9.57 (8.32, 10.85) | 4.81 (3.69, 5.97) | 0.53 (0.24, 0.93) |
| Processed Meat Intake |  |  |  |  |  |
| Never | 9,462 (10%) | 8.70 (8.03, 9.48) | 9.17 (7.94, 10.34) | 5.16 (4.11, 6.30) | 0.63 (0.32, 1.05) |
| <1 time/week | 30,580 (33%) | 8.77 (8.11, 9.55) | 9.31 (8.11, 10.48) | 5.01 (3.99, 6.13) | 0.53 (0.26, 0.92) |
| 1 time/ week | 26,623 (28%) | 8.75 (8.06, 9.52) | 9.40 (8.20, 10.59) | 4.92 (3.91, 6.08) | 0.55 (0.26, 0.95) |
| 2+ times/week | 27,116 (29%) | 8.68 (7.98, 9.49) | 9.61 (8.39, 10.85) | 4.74 (3.69, 5.91) | 0.56 (0.27, 0.97) |
